# Home- and Community-Based Services Spending and Living Arrangements Among Older Adults

**DOI:** 10.1001/jamahealthforum.2026.2064

**Published:** 2026-07-02

**Authors:** Gavin J. Schilling, Ferdous Z. Sardar, Cyrus M. Kosar, Momotazur Rahman

**Affiliations:** 1 Central Connecticut State University, New Britain; 2 Department of Health Services, Policy and Practice, Brown University School of Public Health, Providence, Rhode Island

## Abstract

**Importance:**

The US population is aging rapidly, increasing the future demand for assistance with aging in place.

**Objective:**

To examine the association between higher state allocation of long-term care spending to home- and community-based services (HCBS) and the likelihood of group quarters residence, coresidence with adult children, Medicaid enrollment, and migration among older adults with and without independent living difficulties.

**Design, Setting, and Participants:**

This repeated cross-sectional study used state-year fixed-effect models of American Community Survey microdata to analyze how changes in states’ allocation of long-term care spending to HCBS was differentially associated with aging-in-place indicators and Medicaid enrollment among older adults with vs without independent living difficulties. The study was conducted and data were analyzed between October 2025 and April 2026.

**Exposure:**

Share of state’s long-term services and support budget on HCBS from 2008 to 2020.

**Main Outcomes and Measures:**

Medicaid enrollment, group quarters residence, coresidence with adult children, within-state migration, and out-of-state migration.

**Results:**

Of the 7.35 million older adults in the sample, 17.1% had independent living difficulties. Those with independent living difficulties were older (mean [SD] age, 80.44 [8.73] vs 73.38 [6.80]), more likely to be female (66.7% vs 53.9%), less likely to be married (33.9% vs 59.6%), and less likely to be born in the US (83.9% vs 86.3%). Regression results indicated that a 20–percentage point increase in state HCBS share was associated with a 2.6–percentage point lower likelihood of group quarters residence (95% CI, −3.23 to −1.87), 0.8–percentage point lower likelihood of coresidence with an adult child (95% CI, −1.11 to −0.49), a 1.0–percentage point higher likelihood of residential continuity (95% CI, 0.64 to 1.29), 0.8–percentage point lower likelihood of within-state migration (95% CI, −1.08 to −0.43), and 0.2–percentage point lower likelihood of out-of-state migration (95% CI, −0.28 to −0.14) among older adults with independent living difficulties compared with those without.

**Conclusions and Relevance:**

The results of this cross-sectional study suggest that higher shares of HCBS spending are associated with a higher likelihood of aging in place among older adults with independent living difficulties without markedly increasing Medicaid enrollment, supporting the targeted expansion of HCBS services.

## Key Points

**Question:**

Is higher state spending on home- and community-based services (HCBS) associated with better aging-in-place outcomes for older adults with functional limitations?

**Findings:**

In this repeated cross-sectional study of American Community Survey microdata including 7.35 million older adults, older adults with independent living difficulties residing in states that allocated a larger share of long-term services and supports spending to HCBS exhibited a lower likelihood of group quarters residence, residence with adult children, and migration.

**Meaning:**

The study findings suggest that higher shares of HCBS spending allow older adults in the most need of assistance to age in their homes and lessen the need for informal care.

## Teaser

This cross-sectional study examines the association between higher state allocation of long-term care spending to home- and community-based services and the likelihood of group quarters residence, coresidence with adult children, Medicaid enrollment, and migration among older adults with and without independent living difficulties.

## Introduction

Due to the aging and increased life expectancy of the largest US birth cohort, Baby Boomers, older adults are expected to account for 21% of the US population by 2030, up from 12% in 2000.^1,2^ Most older adults prefer to remain in their own homes and communities, remain autonomous, maintain community-based social ties, and avoid the negative stigma associated with institutional living, (ie, aging in place).^3^ However, changes in cognitive and functional status during the aging process are associated with an increased risk of dementia and relocating and entering institutional care,^4,5,6^ or living with adult children.^7,8^ These risks may be mitigated through high-quality long-term care.^9^ Over the last 3 decades, Medicaid, the largest payer for long-term care,^10,11^ has steadily increased the share of long-term services and supports (LTSS) spending directed to home- and community-based services (HCBS), a subcategory of LTSS, with the explicit aim of expanding community alternatives to nursing homes and supporting older adults’ strong preference to age in place rather than in institutional settings.^12,13,14,15^

Past research has shown that higher state or program HCBS spending is associated with reduced nursing home entry,^14^ long-term nursing home use,^16^ and the likelihood of patients after receiving short-term care becoming a long-stay resident.^13^ Past research has also found that increases in state HCBS spending compared with total LTSS spending were associated with increases in the HCBS workforce.^17,18^ Over the same period, informal and formal home care rose among older adults with disabilities,^7,19^ which was consistent with policy-mediated substitution or complementarity between paid services and family support. Despite these advances, little is known about how state HCBS spending is associated with living arrangements, such as coresidence with adult children or residential relocation. In addition, the effects of HCBS on individuals at high risk for residential changes, namely those with independent living difficulties, have been understudied.

We addressed these gaps by examining American Community Survey (ACS) microdata from 2009 through 2021 to assess how changing the allocation of LTSS spending to HCBS is associated with 4 outcomes central to community living for adults with and without independent living difficulty. We focused on differential associations for those with independent living difficulty because LTSS is most salient for this group, and associations among those without limitations may reflect unobserved geographic factors. We hypothesized that because higher HCBS shares may lower the effective cost of remaining at home for adults with functional impairment, the risk of group quarters residence, coresidence with caregivers, and residential relocation would be reduced. We also examined changes in Medicaid enrollment, hypothesizing that potential changes to Medicaid enrollment as HCBS becomes more available (ie, woodwork effects) would be larger for adults with independent living difficulty.

## Methods

### Study Oversight and Reporting Guideline

This study used deidentified, publicly available data and required no collection of data from human participants. Because the study relied entirely on deidentified, public use microdata, the requirement for informed consent was waived. We reported this study according to the Strengthening the Reporting of Observational Studies in Epidemiology (STROBE) reporting guideline for observational studies.

### Data Sources and Study Sample

We use annual ACS^20^ microdata from 2009 through 2021, which contain information comparable with the decennial census at the person-year level. Each year, approximately 3.5 million addresses across the US and Puerto Rico are sampled. Over the study period, ACS response rates ranged from 71.2% to 97.9% (mean of 91.9%).^21^ We restricted the sample to adults aged 65 years and older.

### Outcomes

The study outcomes were Medicaid enrollment and 4 outcomes pertaining to community-based living, all measured with binary indicators. The community-based living measures were (1) residence in group quarters (institutional or noninstitutional); (2) coresidence with 1 or more adult children (biological, adopted, or stepchildren); (3) out-of-state migration during the previous year; and (4) within-state migration during the previous year. Group quarters residence was used because the ACS does separately identify institutional from noninstitutional group quarters residence. Migration was measured using the ACS item on residence 1 year ago, and we distinguished respondents who moved across vs within states based on differences in residential states.

### Explanatory Variables

Our policy measure was the share of each state’s Medicaid LTSS budget that was devoted to HCBS. These data were compiled from historical LTSS expenditure reports published by the US Centers for Medicare & Medicaid Services on Medicaid.gov, with data beginning as early as 1981.^22^ The Medicaid enrollment ACS item only started in 2009, so we began the analysis then. For each ACS respondent observed in year *t*, we assigned the HCBS share using the state of residence 1 year earlier (from the ACS residence item), which differed from the current state only for movers. This alignment ensured that policy exposure preceded outcomes in year *t* and avoided mechanically assigning a postmove policy to premove decisions. To allow for nonlinearity and ease interpretation, we categorized the continuous HCBS share into 6 groups: 34% or less, 35% to 44%, 45% to 54%, 55% to 64%, 65% to 74%, and 75% or greater.

The primary individual-level regressor is an indicator for independent living difficulty, coded from the ACS disability item: “Because of a physical, mental, or emotional condition, does this person have difficulty doing errands alone such as visiting a doctor’s office or shopping?”^20^ Prior research has shown that many diagnoses are captured within each of the 6 broad ACS disability indicators.^23^ We focused on the independent living difficulty indicator because it summarizes whether functional limitations constrain independent community living. The covariates used in adjusted analyses were individuals’ demographic characteristics, including age, sex, marital status, race, Hispanic ethnicity, educational attainment (measured by the highest year of schooling completed), veteran status, and whether they were born in the US. Race and Hispanic ethnicity were self-reported by ACS respondents using categories defined by the US Census Bureau. These variables were included as covariates to account for demographic differences in aging-in-place outcomes and Medicaid eligibility that may vary by race and ethnicity.

### Statistical Analysis

We first examined the trends in the share of older adults with independent living difficulties. Because of demographic shifts and increases in income and educational attainment among older adults,^2^ we also estimated the share of older adults with independent living difficulties after adjusting for the covariates described previously. To understand the difference in the outcomes of interest between individuals with and without independent living difficulties, we plotted these rates for each group by year.

For inference, we estimated linear probability models in which each outcome was regressed on interactions between an indicator for independent living difficulty and the state’s HCBS share. We first used the categorical version of HCBS share, so the interaction coefficients indicated the excess probability of the outcome for adults with difficulty (vs without) at each HCBS tier relative to the 34% or less reference tier. Our model included state-year fixed effects to capture any common shocks in a state over time (including HCBS spending), state by independent living difficulty fixed effects to absorb time-invariant differences by difficulty status within states, and individual-level covariates. Accordingly, the interaction terms reflected the association between the HCBS share and difference in outcomes between older adults with and without independent living difficulty within the same state-year rather than as a simple before and after HCBS effect for adults with difficulty alone. Therefore, this design relied on the assumption that, conditional on state-year fixed effects, state-difficulty fixed effects, and observed covariates, changes in HCBS share are not associated with other unobserved state-time factors that differentially affect older adults with independent living difficulties compared with without.

For a simpler interpretation, we replaced the categories with a continuous HCBS share that was scaled by 20 percentage points; coefficients then represented the change in the disability gap associated with a 20–percentage point increase in HCBS share. We chose a 20–percentage point benchmark to present a sizable but observed shift in HCBS share; across states with nonmissing HCBS share values in 2008 and 2020, the upper quartile of state-level changes was 19.2 percentage points (eTable 4 in Supplement 1). We also conducted prespecified stratified analyses by marital status, nativity, and sex because the mechanisms creating the association between HCBS generosity and community living differ systematically across these groups. We expected larger associations among unmarried adults, US-born adults, and women given their greater exposure to disability-related residential risks and differences in access to informal caregiving resources. As a robustness check, we re-estimated all models, excluding 2020 to 2021 and in specifications that additionally accounted for interactions of Medicaid expansion and overall Medicaid LTSS spending with the presence of independent living difficulty.

All models applied ACS person weights. All analyses were conducted in Stata, version 18 (StataCorp). The 95% CIs were based on robust standard errors clustered by state. Statistical significance was set at 5%. The HCBS share is missing for California, Delaware, Illinois, and Virginia in 2019 and for Texas and Virginia in 2020. Observations from those state-years were excluded from analyses using HCBS share because exposure could not be assigned.

## Results

Table 1 summarizes the characteristics of older adults in the sample. Individuals reporting independent living difficulty constituted roughly 1 in 6 person-years (100.9 million of 604 million weighted observations) and were older than those without difficulties (mean [SD] age, 80.4 [6.8] vs 73.4 [8.7] years). They were substantially less likely to be married (33.9% vs 59.6%) and have lower levels of educational attainment, with 27.4% lacking a high school diploma compared with 12.5% among those without difficulty. Women and people of racial and ethnic minority groups were represented more in the group with difficulty in living independently, reinforcing the relevance of controls for demographic composition.

**Table 1.** Characteristics of Older Adults in the US Between 2009 and 2021 by Independent Living Difficulty Status

Characteristic	Independent living difficulty, %	
	Individuals without	Individuals with
Total, No.	6 087 469	1 260 731
Weighted total, No.	503 215 152	100 933 209
Age, mean (SD), y	73.38 (6.80)	80.44 (8.73)
Sex		
Female	53.9	66.7
Male	46.1	33.3
Married	59.6	33.9
Race		
Black	83.6	79.1
White	8.4	11.7
Other^a^	8.0	9.3
Hispanic ethnicity	7.4	9.1
Born in the US	86.3	83.9
Education level		
Less than high school	12.5	27.4
High school	40.3	43.7
Some college	19.2	14.0
College graduate or greater	28.0	14.9
Veteran	19.7	15.8

^a^ Includes American Indian or Alaska Native, Asian, Native Hawaiian or Other Pacific Islander, and individuals reporting being multiracial.

**Figure 1.**
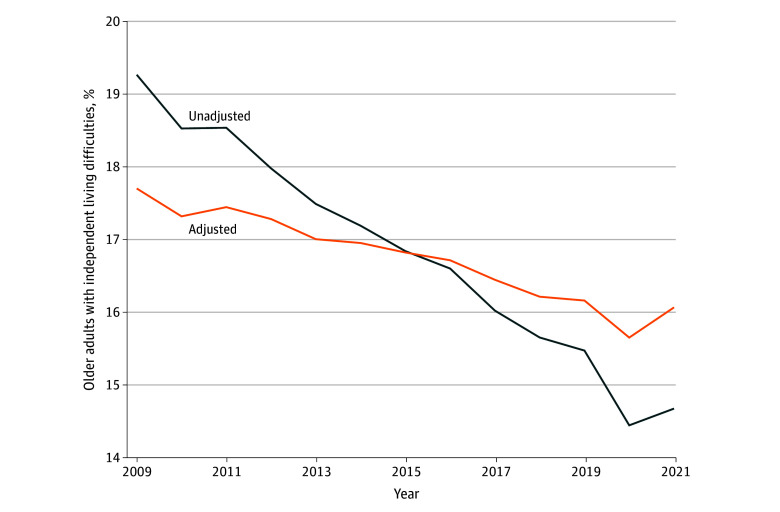
Line Graphs of Trends in the Share of Older Adults in the US With Independent Living Difficulties Adjusted rates are calculated using a regression of the indicator of independent living difficulty onto year indicators controlling for all the covariates listed in the Explanatory Variables subsection of the Methods section and state fixed effects. The rates plotted in the graph are the predicted margins of the year indicators.

Figure 1 shows that the share of older adults in the US with independent living difficulties declined from around 19% in 2009 to about 15% in 2021. Because this decline can be partially explained by demographic shifts, including the entry of Baby Boomers into the study population in later years and increases in education and income levels (eTable 1 in Supplement 1), we also plotted the share after adjusting for control variables (see the regression model used for adjustment in eTable 2 in Supplement 1). The adjusted share of individuals with independent living difficulties declined by about 2 percentage points during the study years.

**Figure 2.**
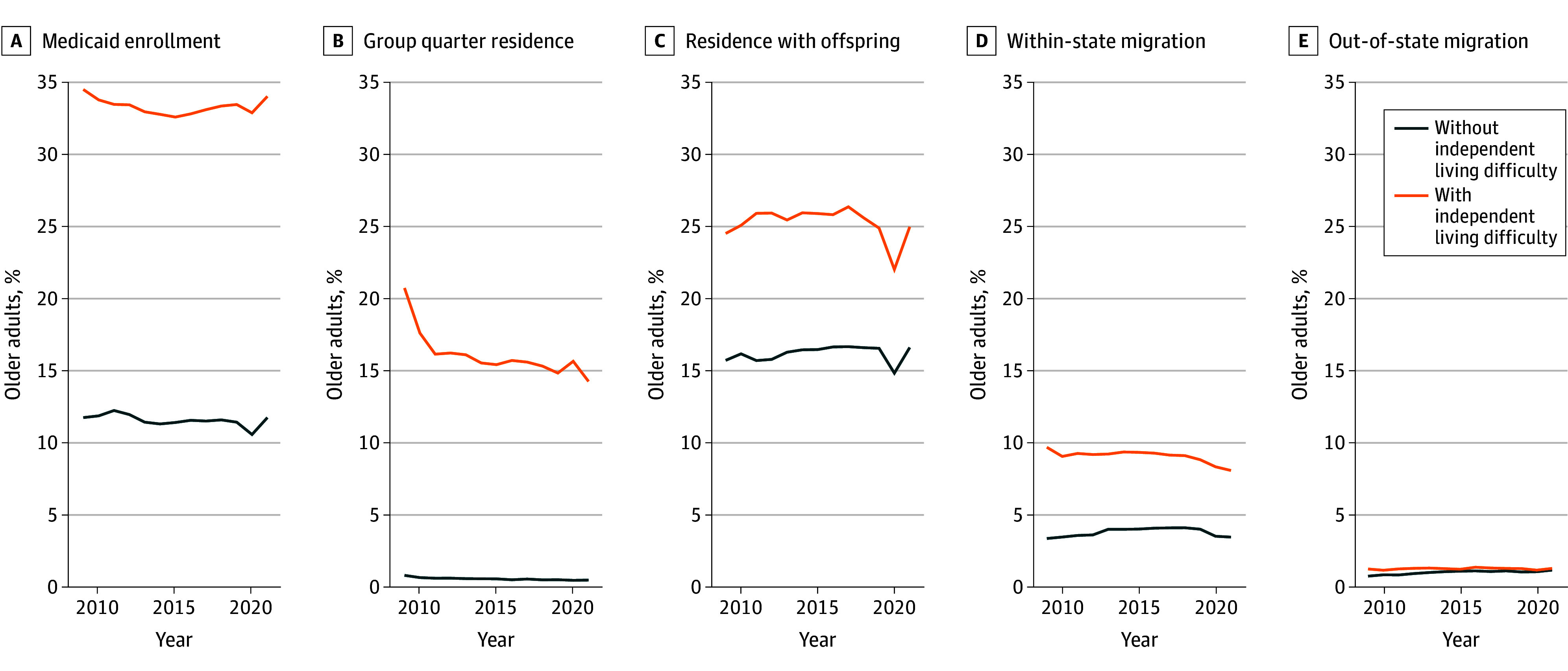
Line Graphs of Aging-in-Place Outcomes Among Older Adults in the US Between 2009 and 2021 by Independent Living Difficulty Status

From 2009 to 2021, adults with independent living difficulty had higher Medicaid enrollment, greater group quarters residence, more frequent coresidence with adult children, and higher within-state migration than those without difficulties (Figure 2). The magnitude of the difference in outcomes between the 2 groups was about constant throughout the study period. Medicaid enrollment was about 22 percentage points higher for persons with independent living difficulties; group quarters residence was about 15 percentage points higher, coresidence with adult children about 9 percentage points higher, and within-state and out-of-state migration about 5 and 0.3 percentage points higher, respectively.

**Figure 3.**
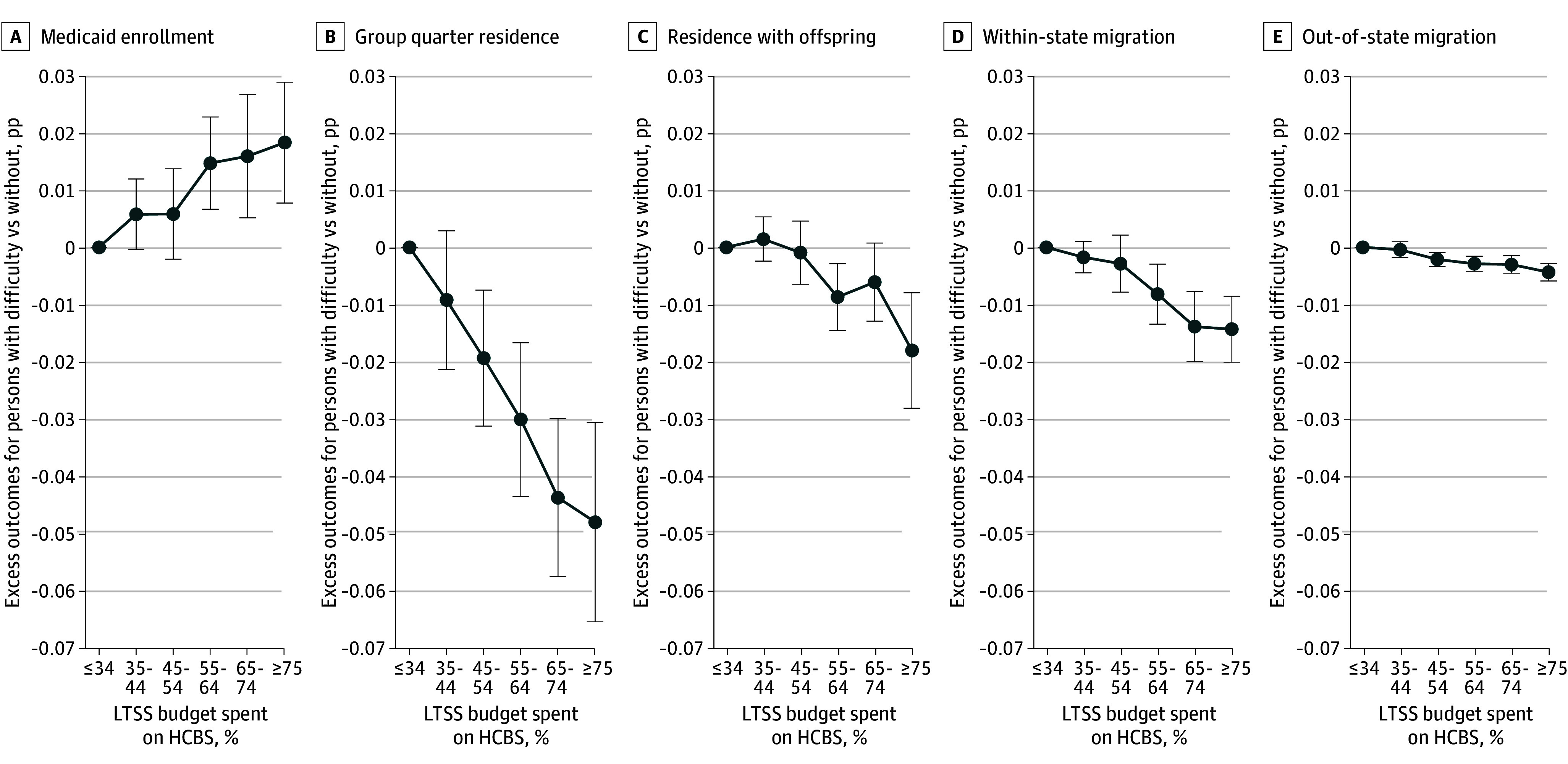
Box and Whisker Plots in Aging-in-Place Outcomes Across Independent Living Difficulty Status by Home- and Community-Based Services (HCBS) Spending Share All regressions include state–difficulty status fixed effects, state-year fixed effects, and control variables. 95% CIs are obtained clustering errors by state. Error bars/whiskers indicate 95% CIs. LTSS indicates long-term services and supports; pp, percentage points.

Because our main specification leveraged within-state year differences between adults with and without independent living difficulties, the regression results described later are best interpreted as showing whether these disability-related gaps were smaller in states with higher HCBS spending shares. Figure 3 shows how the difference in each outcome between older adults with and without independent living difficulties varied across HCBS spending categories, using an HCBS share of 34% or less as the reference category (see the distribution of these categories across the population in eTable 3 in Supplement 1). Because the 75% or greater HCBS share category was relatively sparse overall and concentrated more in later years, the categorical estimates should be interpreted primarily as a descriptive check on the shape of the association. Therefore, we placed greater interpretive weight on the continuous HCBS share specification reported in Table 2. The likelihood of group quarters residence among adults with independent living difficulty was 4.8 percentage points (95% CI, −6.56 to −0.31) lower in states with an HCBS share of 75% or greater than states with an HCBS share of 34% or less. Similarly, compared with low-HCBS share states, individuals with independent living difficulty in high HCBS share states experienced a lower likelihood of coresidence with adult children (−1.81 percentage points; 95% CI, −2.82 to −0.80), within-state migration (−1.44 percentage points; 95% CI, −2.01 to −0.86) and out-of-state migration (−0.44 percentage points; 95% CI, −0.60 to −0.28). These patterns were generally monotonic across HCBS spending categories and indicated that greater HCBS generosity was associated with reduced reliance on institutional settings, intergenerational coresidence, and residential mobility among adults with functional limitations.

**Table 2.** Heterogeneity and Robustness in the Association Between Home- and Community-Based Services Share and Outcome Gaps by Independent Living Difficulty^a^

Characteristic	Difference, percentage points (95% CI)					
	Medicaid enrollment	Residence		Residential continuity	Migration	
		Group quarter	With adult children		Out of state	Within state
Average outcome, %						
Without difficulties	11.59	0.66	16.23	94.99	1.12	3.89
With difficulties	33.22	16.08	25.26	89.51	1.36	9.13
Entire sample	0.84 (0.20 to 1.48)^b^	−2.55 (−3.2 to −1.87)^c^	−0.80 (−1.11 to −0.49)^c^	0.96 (0.64 to 1.29)^c^	−0.21 (−0.28 to −0.14)^c^	−0.752 (−1.08 to −0.43)^c^
Marital status						
Not married	1.25 (0.50 to 2.00)^c^	−2.28 (−3.10 to −1.46)^c^	−0.48 (−0.90 to −0.06)^b^	0.75 (0.34 to 1.15)^c^	−0.19 (−0.30 to −0.09)^c^	−0.55 (−0.93 to −0.178)^c^
Married	0.58 (−0.32 to 1.47)	−1.830 (−2.34 to −1.33)^c^	−0.587 (−1.130 to −0.043)^b^	0.945 (0.614 to 1.280)^c^	−0.165 (−0.286 to −0.044)^c^	−0.780 (−1.110 to −0.446)^c^
Nativity						
Born outside the US	−0.68 (−2.47 to 0.01)	−2.53 (−3.61 to −1.44)^c^	−0.11 (−0.91 to 0.07)	0.73 (0.20 to 1.26)^c^	−0.13 (−0.41 to 0.02)	−0.54 (−1.02 to −0.01)^b^
Born in the US	0.86 (0.29 to 1.42)^c^	−2.30 (−2.93 to −1.65)^c^	−0.94 (−1.30 to −0.58)^c^	0.87 (0.48 to 1.25)^c^	−0.21 (−0.28 to −0.13)^c^	−0.66 (−1.04 to −0.28)^c^
Sex						
Female	0.51 (−0.14 to 1.17)	−3.04 (−3.87 to −2.20)^c^	−0.58 (−0.98 to −0.17)^c^	0.88 (0.51 to 1.24)^c^	−0.23 (−0.32 to −0.13)^c^	−0.65 (−1.02 to −0.28)^c^
Male	1.57 (0.65 to 2.50)^c^	−1.49 (−2.22 to −0.76)^c^	−1.00 (−1.46 to −0.54)^c^	1.21 (0.78 to 1.64)^c^	−0.20 (−0.35 to −0.05)^b^	−1.01 (−1.47 to −0.55)^c^
Estimation excluding 2020 and 2021	0.29 (−0.25 to 0.83)	−2.67 (−3.48 to −1.86)^c^	−0.46 (−0.82 to −0.09)^b^	0.837 (0.354 to 1.320)^c^	−0.18 (−0.28 to −0.08)^c^	−0.66 (−1.14 to −0.18)^c^
Adding Medicaid expansion	0.47 (−0.18 to 1.12)	−2.28 (−3.03 to −1.54)^c^	−0.61 (−1.08 to −0.136)^b^	0.84 (0.45 to 1.23)^c^	−0.18 (−0.28 to −0.07)^c^	−0.66 (−1.03 to −0.29)^c^
Adding LTSS	0.64 (0.10 to 1.18)^b^	−2.29 (−3.03 to −1.54)^c^	−0.75 (−1.08 to −0.42)^c^	0.72 (0.35 to 1.09)^c^	−0.143 (−0.23 to −0.06)^c^	−0.57 (−0.93 to −0.22)^c^

Abbreviations: HCBS, home- and community-based services; LTSS, long-term services and supports.

^a^ All regressions include state–difficulty-status fixed effects, state-year fixed effects, and individual-level control variables. HCBS share is scaled so each coefficient reflects a 20–percentage point increase in HCBS share of LTSS spending. Standard errors are clustered at the state level.

^b^ *P* < .05.

^c^ *P* < .01.

The regression estimates with the continuous HCBS share variable are shown in Table 2 and exhibited the same patterns. A 20–percentage point increase in state HCBS share was associated with a 2.6–percentage point lower likelihood of group quarters residence, a 0.8–percentage point lower likelihood of coresidence with an adult child, 0.8–percentage point lower likelihood of within-state migration, 0.2–percentage point lower likelihood of out-of-state migration, and roughly 1.0–percentage point increase in residential continuity for individuals with independent living difficulties compared with those without. Compared with the baseline disability gaps shown in Table 2, the estimated 2.55–percentage point reduction in group quarters residence corresponded to about 16.5% of the baseline gap, while the estimated reductions in coresidence with an adult child and within-state migration corresponded to about 8.8% and 14.4% of their respective baseline gaps. The overall patterns were broadly consistent across demographic groups, with some variation in point estimate magnitude. For instance, the inverse association of HCBS spending and group quarter residence was even larger among women, and the associations with migration and coresidence with adult children were much higher among US-born older adults than older adults born outside of the US. Sensitivity analyses confirmed that the principal patterns in the full sample results were not associated with the unusual conditions of 2020 and 2021. These patterns were also substantively similar in models that additionally accounted for Medicaid expansion status and overall Medicaid LTSS spending.

Figure 3 shows that compared with low-HCBS share states, individuals with independent living difficulties experienced a 1.83–percentage point (95% CI, 0.76 to 2.92) higher likelihood of Medicaid enrollment in states with an HCBS share of 75% or greater. We observed a directionally consistent result when using the continuous HCBS share (Table 2). Increases in the Medicaid enrollment were concentrated among unmarried, male, and US-born adults, with no clear changes observed for several other groups. The sensitivity analysis that excluded the 2020 and 2021 ACS waves yielded a much smaller and statistically insignificant estimate.

## Discussion

This cross-sectional study examined whether states that allocate a larger share of their Medicaid LTSS budget to HCBS exhibit narrower disparities in aging-in-place outcomes between adults with and without independent living difficulty. We found consistent evidence that higher HCBS spending is associated with reductions in group quarters residence among adults with functional limitations. The estimates also showed modest but meaningful declines in coresidence with adult children and lower probabilities of within-state and out-of-state migration, suggesting greater residential stability in high-HCBS states. These patterns were strongest at the upper end of the HCBS distribution and concentrated among unmarried individuals and US-born adults. We found some evidence of a woodwork effect (ie, enrollment in Medicaid as HCBS becomes more available), but the results were less robust to subgroup and sensitivity analyses.

Our findings aligned with prior work that showed that states that invested more heavily in HCBS had reduced institutional use among older adults.^7,8,9^ These previous studies documented lower nursing home entry in states with higher HCBS spending, and our group quarters results extended this evidence by demonstrating similar patterns outside the formal long-term care system. However, this literature has largely focused on average utilization outcomes or HCBS recipients, providing limited evidence on whether HCBS rebalancing narrows disparities between older adults with and without functional limitations. By examining disability-related gaps in group quarters residence using population-representative ACS data, our findings extend prior work by showing how the effects of higher HCBS spending are more concentrated on older adults with independent living difficulties.

Our study further contributed by examining living arrangements and residential mobility outcomes that are central to aging in place but have been largely excluded from past work.^1,10^ Our results suggest that HCBS rebalancing may reduce the extent to which older adults with functional limitations must rely on family housing or geographic relocation to obtain care. We also contributed to the debate about whether expanding HCBS induces substantial new Medicaid participation among older adults. Although higher HCBS spending shares were associated with a statistically significant increase in the Medicaid enrollment gap, the magnitude was modest and not consistent across the set of analyses. This result was consistent with prior work that found limited evidence of a large woodwork effect in Medicaid enrollment due to HCBS.

### Limitations

This study had limitations. Although this study used a large, nationally representative sample with state-year fixed effects, the results were associational. Because identification relied on within-state–year variation in disability-related outcome gaps, the estimates may still reflect unobserved state-time factors that differentially affect older adults with and without independent living difficulty. Additional specifications that incorporated Medicaid expansion and overall LTSS spending were reassuring but did not eliminate this concern. The policy measure was aggregated at the state level and did not capture variation in program availability, access, or intensity within states, nor did it distinguish effects across specific waiver types, policies (such as the Balancing Incentive Program), or population subgroups. In addition, the migration measures identified whether older adults moved but not the reasons for relocation, making it difficult to infer whether mobility reflects care needs, family dynamics, or other social or economic pressures. Finally, the analysis did not assess program costs or long-term fiscal effects, an important consideration for Medicaid policy given concerns about sustainability and population aging. Future research should use administrative or linked survey claims data to better isolate causal mechanisms, evaluate program heterogeneity, and examine whether reductions in institutionalization and mobility offset the marginal spending required to expand HCBS.

### Conclusions

Taken together, the findings of this cross-sectional study suggest that HCBS rebalancing supports residential continuity. Because institutional entry, migration, and multigenerational coresidence impose costs on families and Medicaid programs, HCBS investments may generate fiscal and social returns beyond the services themselves. The concentration of effects among unmarried and US-born adults highlights a dimension rarely quantified in policy evaluation. These results underscore the importance of prioritizing HCBS capacity, workforce development, and equitable access to noninstitutional care in state Medicaid programs.

## Notes

Supplement 1. eTable 1. Characteristics of the older adult population in the US in 2009 and 2021 eTable 2. Regression for estimation of adjusted share of individuals with independent living difficulties eTable 3. Distribution of HCBS spending share by year eTable 4. Distribution of state HCBS share and state-level change, 2008 to 2020

Supplement 2. Data sharing statement
