# Supplementary material for: Home- and Community-Based Services Spending and Living Arrangements Among Older Adults
Source: JAMA Health Forum. 2026 Jul 2;7(7):e262064. doi: 10.1001/jamahealthforum.2026.2064 (PMC13329704; doi:10.1001/jamahealthforum.2026.2064)
Supplement: Supplement 1. — eTable 1. Characteristics of the older adult population in the US in 2009 and 2021 eTable 2. Regression for estimation of adjusted share of individuals with independent living difficulties eTable 3. Distribution of HCBS spending share by year eTable 4. Distribution of state HCBS share and state-level change, 2008 to 2020 [file jamahealthforum-e262064-s001.pdf]

Supplemental Online Content

Schilling GJ, Sardar FZ, Kosar CM, Rahman M. Home and community-based services spending and living arrangements among older adults. *JAMA Health Forum*. 2026;7(7):e262064. doi:10.1001/jamahealthforum.2026.2064

eTable 1. Characteristics of the older adult population in the US in 2009 and 2021
eTable 2. Regression for estimation of adjusted share of individuals with independent living difficulties
eTable 3. Distribution of HCBS spending share by year
eTable 4. Distribution of state HCBS share and state-level change, 2008 to 2020

This supplemental material has been provided by the authors to give readers additional information about their work.

© 2026 Schilling GJ et al. *JAMA Health Forum*.

eTable 1: Characteristics of the older adult population in the US in 2009 and 2021

	2009	2021
N	478,850	645,002
Weighted N	39,372,307	50,475,283
Age, mean	75.2	74.1
Female	57.5%	54.9%
Married	53.9%	56.3%
Race: White	85.3%	76.6%
Race: Black	8.5%	9.0%
Race: other	6.2%	14.4%
Hispanic	6.8%	8.0%
Born in the US	87.0%	84.7%
Education: less than High School	20.9%	10.2%
Education: high school	43.0%	39.3%
Education: some college	15.8%	20.2%
Education: college graduate or over	20.2%	30.3%
Veteran	22.9%	14.4%

© 2026 Schilling GJ et al. *JAMA Health Forum*.

eTable 2: Regression for estimation of adjusted share of individuals with independent living difficulties

```
. reghdfe indep_liv_dif i.year [w=perwt] , absorb(statefip female veteran educ us_born
age marst race  hispan)
(frequency weights assumed)
(MWFE estimator converged in 9 iterations)

HDFE Linear regression                              Number of obs   =  604148361
Absorbing 9 HDFE groups                             F(  12, 6.0e+08)=   14022.94
                                                    Prob > F        =     0.0000
                                                    R-squared       =     0.1734
                                                    Adj R-squared   =     0.1734
                                                    Within R-sq.    =     0.0003
                                                    Root MSE        =     0.3392

------------------------------------------------------------------------------
indep_liv_~f | Coefficient  Std. err.      t    P>|t|     [95% conf. interval]
-------------+----------------------------------------------------------------
        year |
       2009  |  -.0038382    .000076   -50.51   0.000    -.0039872   -.0036893
       2010  |  -.0025698   .0000756   -34.00   0.000    -.0027179   -.0024216
       2011  |  -.0042075   .0000749   -56.21   0.000    -.0043542   -.0040608
       2012  |  -.0069862   .0000743   -94.03   0.000    -.0071318   -.0068406
       2013  |    -.00751   .0000737  -101.83   0.000    -.0076546   -.0073655
       2014  |  -.0088219   .0000733  -120.42   0.000    -.0089655   -.0086784
       2015  |  -.0098902   .0000728  -135.88   0.000    -.0100328   -.0097475
       2016  |  -.0125784   .0000723  -173.89   0.000    -.0127202   -.0124366
       2017  |  -.0149088   .0000719  -207.33   0.000    -.0150498   -.0147679
       2018  |  -.0154382   .0000715  -215.98   0.000    -.0155783   -.0152981
       2019  |  -.0205432   .0000746  -275.37   0.000    -.0206894    -.020397
       2020  |  -.0163862    .000073  -224.61   0.000    -.0165292   -.0162433
             |
       _cons |   .1770302   .0000543  3262.12   0.000     .1769239    .1771366
------------------------------------------------------------------------------

Absorbed degrees of freedom:
-----------------------------------------------------+
 Absorbed FE | Categories  - Redundant  = Num. Coefs |
-------------+---------------------------------------|
    statefip |        51           0          51     |
      female |         2           1           1     |
     veteran |         2           1           1    ?|
        educ |        11           1          10    ?|
     us_born |         2           1           1    ?|
         age |        33           1          32    ?|
       marst |         6           1           5    ?|
        race |         9           1           8    ?|
      hispan |         5           1           4    ?|
-----------------------------------------------------+
? = number of redundant parameters may be higher

.
end of do-file

. margins i.year

Adjusted predictions                                   Number of obs = 604148361
Model VCE: OLS

Expression: Linear prediction, predict()

------------------------------------------------------------------------------
```

© 2026 Schilling GJ et al. *JAMA Health Forum*.

year	Margin	Delta-method std. err.	z	P>|z|	[95% conf. interval]	
2008	.1770302	.0000543	3262.12	0.000	.1769239	.1771366
2009	.173192	.0000535	3234.47	0.000	.1730871	.173297
2010	.1744605	.0000529	3296.53	0.000	.1743568	.1745642
2011	.1728227	.0000518	3335.28	0.000	.1727212	.1729243
2012	.170044	.0000509	3341.80	0.000	.1699443	.1701437
2013	.1695202	.00005	3389.18	0.000	.1694222	.1696183
2014	.1682083	.0000492	3417.39	0.000	.1681118	.1683048
2015	.1671401	.0000485	3449.19	0.000	.1670451	.1672351
2016	.1644519	.0000477	3446.98	0.000	.1643584	.1645454
2017	.1621214	.000047	3449.53	0.000	.1620293	.1622135
2018	.1615921	.0000463	3490.96	0.000	.1615014	.1616828
2019	.156487	.0000508	3082.22	0.000	.1563875	.1565865
2020	.160644	.0000483	3324.73	0.000	.1605493	.1607387

© 2026 Schilling GJ et al. *JAMA Health Forum*.

eTable 3: Distribution of HCBS spending share by year

year	<=34	35-44	45-54	55-64	65-74	75+	Total
2008	28.55	28.46	21.01	16.13	5.85	0	100
2009	26.67	27.08	24.1	16.29	3.85	2	100
2010	12.63	33.68	27.45	19.45	4.96	1.83	100
2011	17.29	29.82	27.88	18.69	4.28	2.04	100
2012	13.92	32.15	28.18	19.38	4.32	2.05	100
2013	5.82	32.14	32.15	23.46	4.36	2.05	100
2014	11.07	22.91	19.75	37.66	7.22	1.38	100
2015	11.09	11.51	32.19	20.17	21.25	3.79	100
2016	11.1	11.86	29.36	22.69	21.21	3.77	100
2017	4.64	26.03	17.8	27.36	9.49	14.68	100
2018	10.61	16.38	15.6	30.67	5.28	21.45	100
2019	1.09	22.18	20.8	37.46	8.59	9.88	100
2020	0.98	10.08	30.67	17.2	30.44	10.64	100
Total	11.42	22.87	25.06	23.89	10.49	6.27	100

© 2026 Schilling GJ et al. *JAMA Health Forum*.

eTable 4. Distribution of state HCBS share and state-level change, 2008 to 2020

Measure	HCBS share in 2008	HCBS share in 2020	Change in percentage points
N	49	49	49
Mean	43.6	57.1	13.6
Min	16.6	32	-3.2
25th percentile	34.3	48.6	8.1
Median	42.3	55.7	13
75th percentile	51.5	69.2	19.2
Max	74.7	83.9	37.6

Notes: The table includes 48 states and the District of Columbia, excluding Texas and Virginia due to missing HCBS share values in 2020. HCBS share is defined as the percentage of each state's Medicaid long-term services and supports (LTSS) budget allocated to home and community-based services. Values for 2008 and 2020 are drawn from historical LTSS expenditure reports published by the Centers for Medicare and Medicaid Services (CMS) on Medicaid.gov. The change in HCBS share is calculated as the 2020 value minus the 2008 value for each state. Summary statistics reflect the distribution across the 49 included jurisdictions.

© 2026 Schilling GJ et al. *JAMA Health Forum*.
