# Supplementary material for: Home- and Community-Based Services Spending and Living Arrangements Among Older Adults
Source: JAMA Health Forum. 2026 Jul 2;7(7):e262064. doi: 10.1001/jamahealthforum.2026.2064 (PMC13329704; doi:10.1001/jamahealthforum.2026.2064)
Supplement: Supplement 2. — Data sharing statement [file jamahealthforum-e262064-s002.pdf]

Data Sharing Statement

Schilling. Home and Community-Based Services Spending and Living Arrangements Among Older Adults. *JAMA Health Forum*. Published July 02, 2026.
doi:10.1001/jamahealthforum.2026.2064

Data
Data available: No

Additional Information
Explanation for why data not available: Data is already publicly available
